# The Relationship Between Nonsuicidal Self-injury and Attachment: Protocol for a Systematic Review and Meta-analysis

**DOI:** 10.2196/40808

**Published:** 2023-05-31

**Authors:** Soudeh Aghamohammadi, M Ali Mazaheri, Ladan Fata, Fereshteh Mootabi

**Affiliations:** 1 Department of Clinical Psychology Faculty of Education and Psychology Shahid Beheshti University Tehran Iran; 2 Segal Clinical Center Tehran Iran; 3 Department of Basic Research Family Research Institute Shahid Beheshti University Tehran Iran

**Keywords:** nonsuicidal self-injury, NSSI, attachment, emotion regulation, systematic review, review methodology, search strategy, library science, librarian, self-injury, self-harm, self-injurious, self-destructive, self-mutilation, review protocol, meta-analysis, meta-analyses

## Abstract

**Background:**

The prevalence of nonsuicidal self-injury (NSSI) is a major concern in public health. Two main factors (individual and environmental) cause NSSI. Studies addressing NSSI often consider it as an emotion regulation strategy. Studying NSSI within the framework of attachment theory is reasonable since the capacities to regulate emotion come into existence in the framework of attachment in the first periods of a child’s growth. Primary studies addressing this topic are not frequent, and no systematic review has been conducted.

**Objective:**

This systematic review and meta-analysis protocol aims to investigate the relationship between NSSI and attachment style and finding its relationship based on study design, study type, different types of attachments, and gender.

**Methods:**

All studies on the relationship between NSSI and attachment will be included in this systematic review. We will include observational studies (cross-sectional, cohort, and case control) through searches in electronic databases via PubMed, CINAHL, Embase, Web of Science, ProQuest, Scopus, PsycINFO, and Google Scholar as complementary search. Qualitative studies, case studies, case series, and letters to the editor will be excluded. There will be no language limitation. Moreover, there will be no limitations regarding the study participants’ age, gender, nationality, sexual orientation, and psychological problems. Funnel plots were examined if 10 or more studies are included, and the Begg and Egger statistical tests were used to assess the risk of bias. All similar data will be combined through the “metan” command by Stata statistical package (StataCorp). A fixed-effects or random-effects model, considering methodological similarities or differences, will be selected to determine a combination model.

**Results:**

We will summarize the selection of the eligible studies using a flowchart. The results will be presented in a table of evidence. The results of the meta-analysis will be depicted using diagrams and tables.

**Conclusions:**

It seems necessary to carry out such systematic and comprehensive meta-analysis to present a summary of the published articles in terms of the relationship between NSSI and attachment. The results from this review will be used to improve our knowledge of the role of the upbringing of children and NSSI behavior and will help design appropriate interventions to address NSSI.

**Trial Registration:**

PROSPERO CRD42021226455; https://tinyurl.com/yc77wny8

**International Registered Report Identifier (IRRID):**

PRR1-10.2196/40808

## Introduction

### Background

Nonsuicidal self-injury (NSSI) is defined as a direct [1], repeated and socially unacceptable injury to a person’s body tissue [2], a deliberate destruction of one's own body tissue [3], and without suicidal thoughts [4]. It includes cutting, scratching, or burning the surface of one’s body, as well as hitting with objects, and inflicting direct damage to one’s skin or bones by a person [5]. This is a common character in borderline personality disorder [6], and was one of the initial criteria of borderline personality disorder [7]. The criteria for NSSI were presented in Diagnostic and Statistical Manual of Mental Disorders, Fifth Edition (DSM-5), but it needs further empirical study to be mentioned in the next version of DSM. The criteria for NSSI include the following: the person having intentionally injured surface parts of their own body such as cutting, burning, biting, and scratching skin for at least 5 days over the past year; this act cannot have been culturally influenced [8]; the intentional injury should have been associated with negative emotions and feelings such as pervasive anxiety, stress, anger, and confusion; and this act must have been committed to achieve a specific goal [9].

It usually begins in adolescence, the age of onset has been reported to be between 14 and 16 years [10], and its rate decreases in late adolescence [11]. As a common psychiatric symptom, it puts adolescents at greater risk than other age groups [12]. The prevalence of this disorder in teenagers has been reported 16% [13]. Sex differences are also important in this disorder, which occurs more commonly in girls [14]. The prevalence of NSSI in teenage girls is reported to be 19% [15]. There are also cultural differences in the prevalence of these behaviors. The overall prevalence of NSSI (32.6%) is higher in Asian countries compared with Western countries (19.4%) [16].

According to the prevalence of this disorder in different ages and cultures, there are various predisposing factors based on the results of qualitative research. One of the etiological factors, for instance, is attachment styles [17,18]. Generally, the predisposing factors for the development of NSSI can be divided into individual and environmental factors [19]. Adolescence is a period of physical and psychological growth, and it is important because of changes related to puberty, identity, and independence. This stage is accompanied by important and sudden changes such as physical, sexual, cognitive, emotional, and moral changes, as well as changes in desires, values, and patterns. All these factors together create difficulties for teenagers, and training, support, time, and patience are required to answer their questions or have their problems solved. If teenagers cannot manage and solve their problems, they may turn to inappropriate practices such as NSSI [20].

On the other hand, attachment styles are known to be one of the environmental factors, which play an important role in the onset, formation, and maintenance of NSSI in adolescents [21]. Based on the research results, insecure attachment plays an important role in the tendency of people to NSSI [22]. Unfavorable family conditions in the early years of life have been identified as risk factors for presenting NSSI. There are correlations between NSSI and parental absence [23], poor quality of relationship with parents [24,25], and a lack of social support [26].

Attachment is a deep and enduring emotional bond that connects one person to another across time and space. Attachment is an emotional bond between child and the primary caregiver. The caregiver’s response pattern can help regulate the child’s emotional states so that the child can develop a secure attachment style. On the other hand, an inconsistent response pattern or less responsive caregiving can lead to an insecure attachment pattern that causes emotional dysregulation [21]. Some empirical studies have discussed the relationship between insecure attachment and NSSI. In a sample of adult psychiatric patients, they found that preoccupied attachment was associated with lifelong NSSI, but emotional pain partially moderated the relationship between a dysfunctional attachment pattern and NSSI [27].

A longitudinal study showed that the dysfunctional attachment between adolescents and their mothers is associated with a higher frequency of NSSI, which is mediated by increased rates of confused identity style [28]. The quality of attachment to parents through self-compassion, as self-reported by patients, provides us with insights into how such a disorder can emerge in adolescents. All of these results indicate that an insecure attachment pattern increases the likelihood of engaging in NSSI or can maintain it [29]. Despite the importance of these findings, studies in this field, especially among the adolescent population, are scarce [21]. Therefore, the authors will conduct a systematic review and meta-analysis to investigate the relationship between NSSI and attachment.

### Objectives

The primary goal of this systematic review is to investigate the relationship between NSSI and attachment. The secondary objectives are as follows: (1) examine the relationship between NSSI and attachment according to the study type (interventional and observational studies); (2) examine the relationship between NSSI and attachment according to the study approach (cross-sectional, retrospective, and longitudinal); (3) examine the relationship between NSSI and attachment based on different types of attachment (attachment to the primary caregiver, romantic attachment, attachment to peers, etc); (4) examine the relationship between NSSI and attachment based on gender; (5) examine the relationship between NSSI and attachment based on different age groups; and (6) examine the relationship between NSSI and attachment based on studying the difference in findings (heterogeneity) in this relationship and investigating its potential reasons.

## Methods

### Guidelines and Registration

This study is a systematic review that will be reported based on the recommendations of the PRISMA (Preferred Reporting Items for Systematic Reviews and Meta-Analyses) 2009 checklist. The study protocol was registered with the International Prospective Register of Systematic Reviews (CRD42021226455).

### Eligibility Criteria

#### Study Type

We will include observational studies (cross-sectional, cohort, and case control) that have investigated the relationship between NSSI and attachment. Qualitative studies, case studies, case series, and letters to the editor will be excluded. There will be no limitation regarding the language of the citations.

#### Participants

Patients of all age groups (including children, adolescents, youth, and adults), nationalities, sexual orientations, and psychological disorders will be eligible to be included.

#### Intervention

This study will investigate the relationship between NSSI and attachment. Any study that has defined NSSI with any instrument and has assessed its relationship with at least one of the attachment types, including caregiver attachment, romantic attachment, or peer attachment (by any definition criteria or means) will be eligible. In addition, the studies that have defined NSSI according to the description provided by DSM-5 for NSSI, considering the abovementioned factors, will be eligible.

### Data Sources and Search Strategy

#### Electronic Databases

To identify the relevant literature, Search Syntax is developed for 6 platforms to search in different databases. The search will run in MEDLINE (PubMed), SCIE, SSCI, ESCI, CPCI-S, BKCI-S (Web of Science), Scopus, Embase, PsycInfo, Coronavirus Research Database, Publicly Available Content Database (ProQuest) for thesis and dissertations, and other gray literature.

There will be no language restrictions. There will be no publication date restrictions. Google Scholar will be used as a complementary search. Multimedia Appendix 1 shows the search syntax in each database.

#### Other Sources

In addition to electronic databases, other sources, including conference proceedings, dissertations, and previously published review articles, will be searched. Furthermore, 2 key journals (the first 2 journals with the highest record in Scopus search output) will be searched, and citation searching of the previous review articles as well as the included citations will be performed.

### Data Collection and Analysis

#### Selection

After completing the search in the databases, the outputs will be exported to the Mendeley reference manager (Elsevier). The titles and abstracts will be screened for possible selection. In order to increase productivity between the 2 individuals who perform the screening, a checklist was prepared. This checklist includes a table consisting of 3 to 5 inclusion and exclusion criteria, categorized as “yes,” “no,” and “not reported,” to which the screeners can respond by reading the abstract or the title of the article. The report of the 2 main variables of the study (attachment and NSSI) is necessary in these articles, and the answer to rest of the inclusion criteria can be “yes” or “not reported for inclusion.” This table is shown in Multimedia Appendix 2. Subsequently, the full texts of the preliminary studies likely to be included in this article will be reviewed. Based on the criteria defined in the protocol, 2 researchers will review the full texts. Any inconsistency between the 2 researchers will be resolved through consensus to make the final list of the included studies.

#### Data Extraction

The data extraction process will be accomplished independently by 2 reviewers through the reading of the full texts of the preliminary studies using a data collection form, which comprises the following: (1) study identification data (ie, first author, publication date, journal name, type of the study, study design, sampling method, and location); (2) background data (ie, age, gender, number of participants studied, specific population groups, inclusion and exclusion criteria of the studies, and education level of the participants); and (3) data related to the primary objective of the systematic review (ie, the relationship between NSSI and attachment) as well as the secondary objectives (ie, the relationship between NSSI and attachment based on study type, study approach, different types of attachment, gender, and age groups). In order to select the appropriate effect size, we will use correlation coefficient and mean difference or standardized mean difference.

We will use the sample size as well as the mean and standard deviation of the independent variable or its subsets in NSSI and non-NSSI groups in those studies that have not reported the desired effect size measures directly. In addition, in case the required data are depicted in graphs, these will be converted to numerical data using the web-based WebPlotDigitizer tool (Ankit Rohatgi). Further, when the required data are not reported in the preliminary studies, we will try to acquire the missing data via correspondence with the corresponding authors. If the required data cannot be acquired after 3 contacts via email, that particular study will be excluded. Two reviewers will be responsible for the data extraction process separately. In case of any inconsistency between the reviewers, this will be resolved by consensus.

#### Data Analysis

The relative data of one of the effect size measures mentioned earlier will be combined through the “metan” command by Stata, version 13.0 (StataCorp). In order to determine a combination model, a fixed-effects or random-effects model—considering the methodological similarities or differences—will be selected.

### Quality Assessment

The quality assessment of the included studies will be performed using the Joanna Briggs Institute checklist. Two reviewers will conduct this process separately. Then, the agreement between the 2 reviewers will be assessed, and in case of inconsistency, consensus will be used.

### Heterogeneity Assessment

To assess the heterogeneity between the primary studies, we will use Cochran *Q* test and the inconsistency index (*I*^2^). In addition, heterogeneity classification will be conducted using the classification method proposed by Higgins as follows: 0% to 24.9% (mild heterogeneity), 25% to 49.9% (moderate heterogeneity), 50% to 74.9% (severe heterogeneity), and 75% to 100% (highly severe heterogeneity).

### Subgroup Analysis

Subgroup analysis will be performed based on variables such as age, gender, type of the study, design of the study, quality of the study, different types of attachments, and time periods.

### Publication Bias

In order to assess publication bias, funnel plots (if 10 or more primary studies are included) will be depicted and examined. In addition, the Begg and Egger statistical tests will be used to assess the publication bias. *P* values of <.1 will be considered as considerable publication bias.

Sensitivity analysis will be conducted using the one-out remove method and the “metaninf” command in Stata software. Furthermore, subgroup analysis or meta-regression will be used in order to explore the effect of methodologic quality on the meta-analysis results. In case of considerable difference between high- and low-quality studies, the conclusion will be made using high-quality studies.

### Ethical Considerations

Since this systematic review is limited to the published articles, it does not require ethical approval.

## Results

We will summarize the selection of the eligible studies by a flowchart. The results will be presented in a table of evidence. The results of the meta-analysis will be depicted using diagrams and tables.

## Discussion

We will investigate studies regarding the relationship between NSSI and attachment without any limitations regarding the age, nationality, gender, or psychological problems of the participants. We will analyze heterogeneity sources among the primary studies. Despite increasing attention to this phenomenon in recent years, we have observed the lack of meta-analyses on the relationship between attachment—in particular, various types of attachment—and NSSI. Therefore, it seems necessary to conduct a comprehensive systematic review with meta-analysis to elucidate this relationship in detail. The findings of this review will be used to improve our knowledge of the role of the upbringing of children and NSSI behavior and will help design appropriate interventions to address NSSI. Additionally, this review will provide new evidence for psychologists and health policy makers. The advantage of this review is that we will search the gray literature and will not impose any language restriction.

## Appendix

Multimedia Appendix 1 Search syntax in each database.

Multimedia Appendix 2 Decision of the screening process.

## Abbreviations

DSM: Diagnostic and Statistical Manual of Mental Disorders
NSSI: nonsuicidal self-injury
PRISMA: Preferred Reporting Items for Systematic Reviews and Meta-Analyses
