# Supplementary material for: The Relationship Between Nonsuicidal Self-injury and Attachment: Protocol for a Systematic Review and Meta-analysis
Source: JMIR Res Protoc. 2023 May 31;12:e40808. doi: 10.2196/40808 (PMC10267785; doi:10.2196/40808)
Supplement: Multimedia Appendix 1 [file resprot_v12i1e40808_app1.docx]

**Multimedia Appendix 1. Search syntax in each database**

### To identify relevant literature, Sterategy is developed for 6 platforms to search in different databases. The search will run in MEDLINE (Via PubMed), SCIE, SSCI, ESCI, and CPCI-S (Via WoS), Scopus, Embase, and PsycInfo. For thesis and dissertations and other gray literatures, “Coronavirus Research Database” and “Publicly Available Content Database” (Via ProQuest) is searched. Hand searching was also performed.

**PubMed**

**Date: 22 Feb 2023**

**Search Syntax in PubMed:**

(“Self-Injurious Behavior"[Mesh] OR Self-Injur*[tiab] OR “Self Injur*”[tiab] OR “Self Harm*”[tiab] OR selfharm[tiab] OR self-harm[tiab] OR “Nonsuicidal Self Injur*”[tiab] OR NSSI[tiab] OR “Self-Destructive Behavior*”[tiab] OR “Self Destructive Behavior*”[tiab] OR “Self-Destructive Behaviour*”[tiab] OR “Self Destructive Behaviour*”[tiab] OR “Self Mutilating”[tiab] OR Automutilation[tiab] OR (mutilation[tiab] AND auto[tiab]) OR “self cutting”[tiab] OR “self directed violence”[tiab] OR “self inflicted injur*”[tiab] OR “self wounding”[tiab] OR self-harming[tiab] OR “self-inflicted harm”[tiab] OR “self-inflicted mutilation”[tiab] OR “self-inflicted wound*”[tiab] OR self-mutilative[tiab] OR “selfinflicted injur*”[tiab] OR “selfinjurious behavior*”[tiab] OR selfinjury[tiab] OR selfinjuries[tiab] OR selfmutilation[tiab]) AND ("Object Attachment"[Mesh] OR “Object Attachment*”[tiab] OR “Psychological Bond*”[tiab] OR (Bond*[tiab] AND Psycholog*[tiab]) OR “Object Relation*”[tiab] OR (Relation*[tiab] AND Object[tiab]) OR “Symbiotic Relation*”[tiab] OR “Emotional Bond*”[tiab] OR “emotional attachment”[tiab] OR “psychological attachment”[tiab])

**Embase**

**Date: 22 Feb 2023**

**Search Syntax in Embase**

Row 1: 'self injurious behavior*':ti,ab,kw OR 'self injurious behaviour*':ti,ab,kw OR 'intentional self injur*':ti,ab,kw OR 'intentional self harm':ti,ab,kw OR 'nonsuicidal self injur*':ti,ab,kw OR nssi:ti,ab,kw OR 'deliberate self-harm':ti,ab,kw OR 'deliberate self harm':ti,ab,kw OR 'self injury':ti,ab,kw OR 'non-suicidal self injur*':ti,ab,kw OR 'non suicidal self injur*':ti,ab,kw OR 'self harm':ti,ab,kw OR 'self-destructive behavior*':ti,ab,kw OR 'self destructive behavior*':ti,ab,kw OR 'self mutilating behavior*':ti,ab,kw OR 'self-mutilating behavior*':ti,ab,kw OR 'self-destructive behaviour*':ti,ab,kw OR 'self destructive behaviour*':ti,ab,kw OR 'self mutilating behaviour*':ti,ab,kw OR 'self-mutilating behaviour*':ti,ab,kw OR automutilation:ti,ab,kw OR 'auto mutilation':ti,ab,kw OR 'self cutting':ti,ab,kw OR 'self directed violence':ti,ab,kw OR 'self inflicted injur*':ti,ab,kw OR 'self wounding':ti,ab,kw OR 'self harming':ti,ab,kw OR 'self-inflicted mutilation':ti,ab,kw OR 'self-inflicted wound':ti,ab,kw OR 'self-inflicted wounding':ti,ab,kw OR 'self-injuring behavior*':ti,ab,kw OR 'self-injuring behaviour*':ti,ab,kw OR 'self-inflicted harm':ti,ab,kw OR 'self-injurious behavior*':ti,ab,kw OR 'self-injurious

21183

Row 2: 'automutilation'/exp

23671

Row 3: #1 OR #2

30038

Row4: 'object attachments' OR 'psychological bond*' OR 'object relationship*' OR 'symbiotic relation*' OR 'emotional bond*' OR 'object relation*' OR 'emotional attachment' OR 'mental attachment' OR 'psychological attachment'

30038

Row 5: #3 AND #4

198

**Scopus**

**Date: 22 Feb 2023**

**Search Syntax in Scopus**

TITLE-ABS-KEY ( "Self Injurious Behavior*"  OR  self-injurious  AND behavior*  OR  "Self Injurious Behaviour*"  OR  "Self-Injurious Behaviour*"  OR  "Intentional Self Injur*"  OR  "Intentional Self Harm"  OR  "Nonsuicidal Self Injur*"  OR  nssi  OR  "Deliberate Self-Harm"  OR  "Deliberate Self Harm"  OR  self-injury  OR  "Self Injury"  OR  "Non-Suicidal Self Injur*"  OR  "Non Suicidal Self Injur*"  OR  "Self Harm"  OR  "Self-Destructive Behavior*"  OR  "Self Destructive Behavior*"  OR  "Self Mutilating Behavior*"  OR  "Self-Mutilating Behavior*"  OR  "Self-Destructive Behaviour*"  OR  "Self Destructive Behaviour*"  OR  "Self Mutilating Behaviour*"  OR  "Self-Mutilating Behaviour*"  OR  automutilation  OR  auto-mutilation  OR  "auto mutilation"  OR  "self cutting"  OR  "self directed violence"  OR  "self inflicted injur*"  OR  "self mutilation"  OR  "self wounding"  OR  self-harming  OR  "self-inflicted harm"  OR  "self-inflicted mutilation"  OR  "self-inflicted wound"  OR  "self-inflicted wounding"  OR  "self-injuring behavior*"  OR  "self-injuring behaviour*"  OR  "self-inflicted harm"  OR  "self-injurious behavior*"  OR  "self-injurious behaviour*"  OR  self-mutilation  OR  "self-mutilative behavior*"  OR  "self-mutilative behaviour*"  OR  selfharm  OR  "selfinflicted injur*"  OR  "selfinflicted wound*"  OR  "selfinjuring behavior*"  OR  "selfinjuring behaviour*"  OR  "selfinjurious behavior*"  OR  "selfinjurious behaviour*"  OR  selfinjur*  OR  selfmutilation )  AND  TITLE-ABS-KEY ( "Object Attachments"  OR  "Psychological Bond*"  OR  "Object Relationship*"  OR  "Symbiotic Relation*"  OR  "Emotional Bond*"  OR  "Object Relation*"  OR  "emotional attachment"  OR  "mental attachment"  OR  "psychological attachment" )

**WoS**

**Date: 22 Feb 2023**

**Search Syntax in WoS**

TS=(“Self Injurious Behavior*” OR Self-Injurious Behavior* OR “Self Injurious Behaviour*” OR “Self-Injurious Behaviour*” OR “Intentional Self Injur*” OR “Intentional Self Harm” OR “Nonsuicidal Self Injur*” OR NSSI OR “Deliberate Self-Harm” OR ”Deliberate Self Harm” OR Self-Injury OR “Self Injury” OR “Non-Suicidal Self Injur*” OR “Non Suicidal Self Injur*” OR “Self Harm” OR “Self-Destructive Behavior*” OR “Self Destructive Behavior*” OR “Self Mutilating Behavior*” OR “Self-Mutilating Behavior*” OR “Self-Destructive Behaviour*” OR “Self Destructive Behaviour*” OR “Self Mutilating Behaviour*” OR “Self-Mutilating Behaviour*” OR Automutilation OR auto-mutilation OR “auto mutilation” OR “self cutting” OR “self directed violence” OR “self inflicted injur*” OR “self mutilation” OR “self wounding” OR self-harming OR “self-inflicted harm” OR “self-inflicted mutilation” OR “self-inflicted wound” OR “self-inflicted wounding” OR “self-injuring behavior*” OR “self-injuring behaviour*” OR “self-inflicted harm” OR “self-injurious behavior*” OR “self-injurious behaviour*” OR self-mutilation OR “self-mutilative behavior*” OR “self-mutilative behaviour*” OR selfharm OR “selfinflicted injur*” OR “selfinflicted wound*” OR “selfinjuring behavior*” OR “selfinjuring behaviour*” OR “selfinjurious behavior*” OR “selfinjurious behaviour*” OR selfinjur* OR selfmutilation) AND TS=(“Object Attachments” OR “Psychological Bond” OR “Object Relationship*” OR “Symbiotic Relation*” OR “Emotional Bond*” OR “Object Relation*” OR “emotional attachment” OR “mental attachment” OR “psychological attachment”)

**PsycInfo**

**Date: 22 Feb 2023**

**Search Syntax in PsycInfo**

( SU ( “Self Injurious Behavior*” OR Self-Injurious Behavior* OR “Self Injurious Behaviour*” OR “Self-Injurious Behaviour*” OR “Intentional Self Injur*” OR “Intentional Self Harm” OR “Nonsuicidal Self Injur*” OR NSSI OR “Deliberate Self-Harm” OR ”Deliberate Self Harm” OR Self-Injury OR “Self Injury” OR “Non-Suicidal Self Injur*” OR “Non Suicidal Self Injur*” OR “Self Harm” OR “Self-Destructive Behavior*” OR “Self Destructive Behavior*” OR “Self Mutilating Behavior*” OR “Self-Mutilating Behavior*” OR “Self-Destructive Behaviour*” OR “Self Destructive Behaviour*” OR “Self Mutilating Behaviour*” OR “Self-Mutilating Behaviour*” OR Automutilation OR auto-mutilation OR “auto mutilation” OR “self cutting” OR “self directed violence” OR “self inflicted injur*” OR “self mutilation” OR “self wounding” OR self-harming OR “self-inflicted harm” OR “self-inflicted mutilation” OR “self-inflicted wound” OR “self-inflicted wounding” OR “self-injuring behavior*” OR “self-injuring behaviour*” OR “self-inflicted harm” OR “self-injurious behavior*” OR “self-injurious behaviour*” OR self-mutilation OR “self-mutilative behavior*” OR “self-mutilative behaviour*” OR selfharm OR “selfinflicted injur*” OR “selfinflicted wound*” OR “selfinjuring behavior*” OR “selfinjuring behaviour*” OR “selfinjurious behavior*” OR “selfinjurious behaviour*” OR selfinjur* OR selfmutilation ) OR MA (Automutilation) ) AND ( SU ( “Object Attachments” OR “Psychological Bond*” OR “Object Relationship*” OR “Symbiotic Relation*” OR “Emotional Bond*” OR “Object Relation*” OR “emotional attachment” OR “mental attachment” OR “psychological attachment” ) OR MA object attachement )

**ProQuest**

**Date: 22 Feb2023**

**Search Syntax in ProQuest**

fulltext(“Self Injurious Behavior*” OR Self-Injurious Behavior* OR “Self Injurious Behaviour*” OR “Self-Injurious Behaviour*” OR “Intentional Self Injur*” OR “Intentional Self Harm” OR “Nonsuicidal Self Injur*” OR NSSI OR “Deliberate Self-Harm” OR ”Deliberate Self Harm” OR Self-Injury OR “Self Injury” OR “Non-Suicidal Self Injur*” OR “Non Suicidal Self Injur*” OR “Self Harm” OR “Self-Destructive Behavior*” OR “Self Destructive Behavior*” OR “Self Mutilating Behavior*” OR “Self-Mutilating Behavior*” OR “Self-Destructive Behaviour*” OR “Self Destructive Behaviour*” OR “Self Mutilating Behaviour*” OR “Self-Mutilating Behaviour*” OR Automutilation OR auto-mutilation OR “auto mutilation” OR “self cutting” OR “self directed violence” OR “self inflicted injur*” OR “self mutilation” OR “self wounding” OR self-harming OR “self-inflicted harm” OR “self-inflicted mutilation” OR “self-inflicted wound” OR “self-inflicted wounding” OR “self-injuring behavior*” OR “self-injuring behaviour*” OR “self-inflicted harm” OR “self-injurious behavior*” OR “self-injurious behaviour*” OR self-mutilation OR “self-mutilative behavior*” OR “self-mutilative behaviour*” OR selfharm OR “selfinflicted injur*” OR “selfinflicted wound*” OR “selfinjuring behavior*” OR “selfinjuring behaviour*” OR “selfinjurious behavior*” OR “selfinjurious behaviour*” OR selfinjur* OR selfmutilation) AND fulltext(“Object Attachments” OR “Psychological Bond*” OR “Object Relationship*” OR “Symbiotic Relation*” OR “Emotional Bond*” OR “Object Relation*” OR “emotional attachment” OR “mental attachment” OR “psychological attachment”)
