# Supplementary material for: The Relationship Between Nonsuicidal Self-injury and Attachment: Protocol for a Systematic Review and Meta-analysis
Source: JMIR Res Protoc. 2023 May 31;12:e40808. doi: 10.2196/40808 (PMC10267785; doi:10.2196/40808)
Supplement: Multimedia Appendix 2 [file resprot_v12i1e40808_app2.docx]

**Multimedia Appendix 2.** Decision of the screening process.

| n | Article code | NSSI | Attachment | Study type | Study approach | Final decision |
| --- | --- | --- | --- | --- | --- | --- |
| 1 |  |  |  |  |  |  |
| 2 |  |  |  |  |  |  |
